# A Fraud of Authors

**Published:** 1809-05

**Authors:** 


### *To the Editors of the Medical and Physical Journal.*

Gentlemen,

THROUGH the medium of your useful and widely circulated publication, I could wish to notice a practice which I cannot help thinking deserving reprobation in authors, and which is becoming very prevalent; a sort of jockeyship. A gentleman of some professional celebrity or notoriety, publishes an useful work; it is advertised; well spoken of; and from the laudable desire which the generality of practitioners have of adding to their stock of knowledge, it becomes generally purchased; in a short time a new edition is announced, with considerable additions, improvements, corrected, revised, additional plates, &c. How, think you, do the purchasers of the first edition feel? why, that they have been buying a work, and that perhaps at an expence which they could but just afford; ford; and in the short space of a year, or year and half, it is become of scarcely any value, compared with the second edition, which a more patient and fortunate man waited for. Is this fair to the purchasers of the first edition, who have paid the first compliment to the author, and who by so doing, and by speaking of the work as it merited, (probably) have been the cause of the rapidity with which the first was sold. Is it honourable? is it just?

Ought not the author to add to his new and almost puffing advertisement as follows: Gentlemen possessing first editions, may be accommodated with the additions, improvements, corrections, and additional plates, printed so as to bind up with their first edition, by application to the bookseller, and producing the first edition, at *such a price* (a moderate one), yet such as to pay for the printing.

The late highly and deservedly eminent Dr. Currie, published his valuable Treatise on cold Affusion in Fevers, in one volume. A few years afterwards he published a sesond edition, much improved and enlarged, in two volumes. I wrote to him, and complained; in less than a fortnight appeared this addition to his advertisement: Gentlemen in possession of the first volume may have the second, which contains all the new matter and cases, by applying to the bookseller, and producing the first volume. He instantly saw the propriety, justness, and fairness of the complaint, and acted accordingly, and as he ought. Country practitioners, with very limited incomes, and amongst whom the general diffusion of knowledge is highly necessary and of the utmost utility, feel the unhandsome conduct I complain of very severely; they wish to keep up their early acquired stock; and often with difficulty part with some hard earned money to keep upon a par with their later informed competitors, by purchasing works of this description, and in a short time learn, to their mortification, that they got but a part of what is generally known, and that full of errata.

Now, to a case in point: I am a practitioner of this description, and in *December* 1807, purchased, at the expence of twelve shillings (to me no trifling sum), a work, entitled "First Lines of the Practice of Surgery," a very useful work, and particularly so to men like myself; I also purchased a prize Essay on Diseases of the Joints.

In the short space of fourteen months, I read in a weekly paper [Mirror of the Times], an advertisement, announcing "First Lines of the Practice of Surgery," second edition, *revised, corrected, and enlarged,* with *several additional*

*additional plates*, price 14s. Now, I must either purchase this (by which the work will cost me 1l. 6s.) or be behindhand in the information which I ought to possess.

Pray, Mr. Editors, take up this matter in the behalf of such unfortunate men as myself. I perceive I ought to have waited patiently, but I was anxious to get a little lore, in addition and refreshment to my almost worn out stock: indeed, I feel grievously hurt.

This same gentleman, I see, has published a work, entitled "A Dictionary of Practical Surgery," price 15s. I think from the detail, and from his other productions, it must be an useful work, and feel almost inclined to save up a trifle gradually until I can afford to buy it; but, perhaps, in a short time after I have so done, a new edition in 4to. may be announced, with plates of instruments, &c. at 1l. 1s. or 1l. 10s. yet I am loth to give it up.

I would not thus trespass upon you; but you will deem it a little claim, when I tell you I have been a purchaser of your useful work from the first, and have scraped together enough to have twenty volumes neatly half bound. And lastly, that I am an old but not rich member of the Royal College of Surgeons in London.

---

*** A complaint somewhat similar having appeared in a rival Journal, which we have noticed in our present Number, we could not, without the suspicion of great partiality, refuse a place for the above.

